# *Tella* intake among pregnant and lactating mothers: may it affect child growth?

**DOI:** 10.1017/jns.2022.40

**Published:** 2022-06-06

**Authors:** Zelalem Tafese, Yifru Berhan, Barbara J. Stoecker

**Affiliations:** 1 School of Nutrition, Food Science and Technology, Hawassa University, Hawassa, Ethiopia; 2 St. Paul's Hospital, Millennium Medical College, Addis Ababa, Ethiopia; 3 Department of Nutritional Sciences, Oklahoma State University, Stillwater, OK, USA

**Keywords:** Ethiopia, Fetal growth, Food security, Lactation, Prenatal alcohol exposure, Qualitative data, Stunting

## Abstract

Alcohol abuse among women is a public health importance that may impair prenatal and postnatal growth. *Tella* is among the most common homemade alcoholic beverages in rural Ethiopia, but little is known about the magnitude of *tella* intake during pregnancy and lactation or its effects on child growth. The present study investigated associations between maternal *tella* intake and the growth of their children. A cross-sectional mixed-methods study was conducted with mothers (*n* 228) and their 12–36-month-old children and with key informants (*n* 12). *Tella* intake during most recent pregnancy and lactation was estimated retrospectively by glasses per drinking event and frequency of events. Nearly 80 % of mothers had consumed some amount of *tella* during their most recent pregnancy and lactation. Furthermore, 72 % of children had tasted or drunk *tella* at some time during their life. Stunting was 42 % and was significantly associated with maternal *tella* consumption at least every other day during pregnancy (adjusted odds ratio (AOR) 4⋅97, 95 % confidence interval (CI) 2⋅20, 11⋅25), male sex (AOR 2⋅31, 95 % CI 1⋅27, 4⋅19), two or more under-5-year-old children in the household (AOR 3⋅52, 95 % CI 1⋅49, 8⋅33) and family size >5 (AOR 1⋅84, 95 % CI 1⋅01, 3⋅36). Underweight was 24⋅6 % and was associated with the child drinking *tella* with their mother (AOR 4⋅23, 95 % CI 1⋅99, 8⋅97), being male (AOR 3⋅73, 95 % CI 1⋅73, 7⋅94), having ≥3 diarrhoeal episodes in the last 3 months (AOR 11⋅83, 95 % CI 4⋅22, 33⋅14) and being in the older age group (AOR 2⋅98, 95 % CI 1⋅09, 8⋅13). The associations between *tella* intake and child growth suggest the need to mitigate the effects of *tella* on child anthropometry.

## Introduction

Globally, alcohol intake during pregnancy is a public health concern^(1)^. Although consumption of alcoholic beverages is a risk factor for problems of growth and development of the fetus^(2)^, a considerable number of women consume alcohol during pregnancy^(1)^. In addition to the increased risk of occurrence of spontaneous abortion and preterm delivery among repetitive users of alcohol during pregnancy, there is an increased risk of fetal alcohol syndrome and fetal alcohol spectrum disorders, which are partly manifested by prenatal and postnatal growth deficiency^(3,4)^. Due to the risks associated with alcohol intake during pregnancy, experts in most countries recommend that pregnant women abstain completely from alcohol intake^(5)^. However, policies in many developing countries have given little attention to these risks^(4)^. Locally prepared alcoholic drinks, in particular, may not be considered harmful to pregnancy outcomes. As a result, the burden of alcohol use during pregnancy in some African countries is high^(3,6,7)^.

Some authors identified alcohol consumption as a public health problem due to its wide use and the associated fetal and childhood complications^(8)^, as well as problems related to compromised placental blood flow to the fetus^(9)^. Furthermore, alcohol acts on the brain in different ways, depending on the type of brain cell and embryo-fetal development stage, leading to structural and functional changes, including cell death and impaired new cell formation^(9,10)^.

Alcohol intake during pregnancy has been noted as a factor associated with birth defects^(11,12)^, low birth weight^(13)^ and stillbirth^(14)^. No consensus exists on a safe amount of alcohol that could be ingested during pregnancy, because even low levels of prenatal exposure may negatively affect embryo-fetal growth and development^(15)^. Therefore, national health services in various countries recommend that women completely abstain from using alcohol throughout their pregnancy period^(7,15)^.

Variations in alcohol consumption have been reported among African countries^(6,7,16,17)^. Although several studies found a considerable proportion of pregnant mothers in Ethiopia and elsewhere to consume alcohol^(7,16,17)^, the association of maternal alcohol intake with child growth has not been well addressed.

Ethiopia has a variety of homemade traditional fermented beverages, including *Tella*, *Tej* and *Araki*^(18)^. *Tella*, by far the most common alcoholic beverage in rural Ethiopia, is made from different cereals, including sorghum, *tef*, wheat, barley and maize, and has 2–4 % alcohol content^(19)^. *Tella* is perceived as a popular and harmless social drink that is highly valued by society^(18,19)^. Thus, the primary aim of the study was to better understand the relation between maternal *tella* intake and the nutritional status of their 12–36-month-old children. Additional risk factors that might contribute to undernutrition of young children in communities within Ethiopia's Productive Safety Net Program (PSNP) areas were also investigated.

## Methods

### Study area and design

This retrospective mixed-method community-based cross-sectional study was conducted in the Meket district of the Amhara region from September to October 2019. The district was selected due to the high number of food-insecure households targeted for the development of food security activities. Of the 37 kebeles in the district, 10 kebeles (smallest administrative unit in Ethiopia) were selected randomly. Lists of food-insecure households targeted for PSNP with children in the age range of 12–36 months were prepared in each kebele by local community health workers.

### Quantitative data collection

#### Sample size and sampling procedure

Using a single proportion population formula, a sample size of 231 mothers with children of age 12–36 months was calculated. The number of mother–child pair was proportionally allocated to the kebeles based on the total number of food-insecure households with 12–36-month-old children. Mothers interviewed were selected from the list of appropriate households in each kebele using a random number table. Women who were not permanent residents or who had mental problems would have been excluded but there were none. One child (with their mother) was excluded because the child was being treated for severe acute malnutrition (SAM).

#### Socioeconomic and demographic data collection

Experienced data collectors with bachelor's degrees were recruited from the study area and worked under the close supervision of the lead author. To ensure data quality, training was given for 2 d on the study objectives, the relevance of the study, informed consent, the confidentiality of the information and interviewing techniques. A pilot study was done with a few women to confirm possible local dialect variations and timing for administration. Maternal and household data were collected by face-to-face interviews with each mother at her residence using a pre-tested semi-structured questionnaire. The interview focused on maternal age, income and marital status, estimated average monthly household income, family size, number of under-5-year-old children and details about *tella* consumption as described in the following.

#### Child anthropometric data

Weight was measured without coats, shoes or additional heavy clothing using a calibrated SECA electronic balance with a measuring range of 25 kg (UNICEF Seca 770) and recorded to the nearest 0⋅1 kg^(20)^. Instrument calibration was checked before weighing each child and the weighing scale was tested daily against a standard weight for accuracy. Lengths or heights were measured without shoes using a stadiometer: for 6–23 months, length was taken and for 24–36 months height was recorded to the nearest 0⋅1 cm. Anthropometric classifications were based on global standards^(21)^. Children with length/height-for-age *z*-scores (LH/AZ) and weight-for-age *z*-scores (WAZ) below −2 sd of the median of the reference population were considered as stunted or underweight, respectively^(20)^. Each child's anthropometric status was calculated using the Emergency Nutrition Assessment for Standardized Monitoring and Assessment of Relief and Transitions (ENA for SMART, 2011) software, which utilises the WHO growth standards.

#### Child morbidity and feeding practices

Child illnesses, such as diarrhoea, respiratory tract infections or malaria were recorded as reported by the mother for 1 month prior to the survey. Mothers were also asked if their child had diarrhoea within the last 3 months. Child dietary data focused on the age complementary feeding began and consumption of animal-source foods (ASFs) as well as on child alcohol exposure.

#### *Tella* consumption by mother and child

The data on maternal *tella* intake were collected by asking about *tella* intake in the 24 h before the survey, as well as the frequency of intake during the last pregnancy and lactation periods. Mothers were also asked about the frequency of drinking events, the number of glasses of *tella* usually consumed per drinking event, about their reasons for consuming *tella* and about giving *tella* to their child.

### Qualitative data collection and analysis

Twelve individual key informant (KI) interviews were conducted with eight purposely selected mothers, two agricultural extension workers and two health extension workers from the study area. The number of KI interviews was determined by the need to achieve saturation of information^(22)^. The interviews provided the perception of the community about *tella* and confirmed the data on probable *tella* intake by mothers and children. Additionally, the KIs were asked the main reasons as why community members chose to consume *tella*, the frequency of *tella* preparation at the household level and the estimated proportion of household food crops used for *tella* production. For clarity of the qualitative information and to prevent loss of data, a voice recorder was used during each interview, and notes were taken by the interviewer and the lead author. These data were collected using the Amharic language as a medium. The qualitative data from KI interview transcripts were analysed thematically and triangulated with quantitative findings^(23)^.

### Quantitative data analysis

Stunting and underweight were dependent variables in the logistic analysis. The dichotomous variables for stunting and underweight were defined as 1 for stunted and 0 for not stunted, and 1 for underweight and 0 for not underweight. Quantitative data were checked, coded and analysed using STATA 14 (Stata/se 14) statistical package.

Categorical data were compiled using descriptive statistics (number and percentage), whereas mean and standard deviation were used to present continuous variables. The normal distribution of the data was checked with the Kolmogorov–Smirnov test^(24)^. Bivariate analyses were conducted, and all variables with *P* < 0⋅25 were included in the multivariable logistic regressions. This selection of variables was based on the Wald test for logistic regression. A stepwise backward elimination technique was used to identify determinants of stunting, and underweight separately and adjusted odds ratios (AORs) with a *P* < 0⋅05 and their corresponding 95 % confidence intervals (CIs) were computed; and crude and adjusted odds ratios are reported. The model fit was checked using the Hosmer–Lemeshow goodness of fit test. Multivariable logistic regression analyses were conducted to determine associations of *tella* consumption and of other household and health-related factors with undernutrition. Associations were declared significant at *P* ≤ 0⋅05.

### Ethical consideration

Ethical clearance was secured from the Institutional Review Board (IRB) of Hawassa University College of Medicine and Health Sciences. The study was also reviewed by the IRB at Oklahoma State University. Data were collected after getting permission from the district health office and obtaining informed consent from the eligible participants.

## Results

### Socio-demographic characteristics

From the total 243 proposed study participants, one child who was on SAM treatment and his mother were excluded from the study; complete responses were obtained from 228 respondents making the response rate 93⋅8 %. The mean family size was 4⋅8 (±1⋅5) persons (Table 1) and households with >2 under-5-year-old children were 15 % (data were not shown). The majority of the mothers were housewives 76 %. The mean ( and standard deviation) age of the children was 27⋅2 (sd 5⋅2) months and more than half (53 %) were males (Table 2).

**Table 1.** Socioeconomic and demographic characteristics of mothers and their *tella* consumption patterns, northern Ethiopia, 2019 (*n* 228)

Variables	Frequency (*n*)	Percentage (%)	Mean (sd)
Age, years			30⋅0 (7⋅1)
Family size			4⋅8 (1⋅5)
Children <5 years			1⋅6 (0⋅8)
Marital status			
Not married	13	5⋅7	
Married/living together	188	82⋅5	
Divorced/widowed/separated	27	11⋅8	
Had own income	85	37⋅3	
Education status			
No formal education	171	75⋅0	
Primary school (1–8)	49	21⋅5	
Secondary school and above	8	3⋅5	
Occupation			
Housewife	174	76⋅3	
Farmer	4	1⋅8	
Merchant	3	1⋅3	
Daily laborer/Other employment	47	20⋅6	
Monthly household income (ETB)			
≥4500 ETB	20	8⋅7	
2500–4499 ETB	68	29⋅8	
500–2499 ETB	112	49⋅2	
<500 ETB	28	12⋅3	
*Tella* consumption patterns			
Drank *tella* in last 24 h before survey	152	66⋅7	
One glass	94	41⋅2	
Two or more glasses	58	25⋅4	
Drank *tella* during last pregnancy	179	78⋅5	
At least once in 2 d	94	41⋅2	
At least once in 3–7 d	85	37⋅3	
Drank *tella* during last lactation	184	80⋅7	
At least once in 2 d	74	32⋅5	
At least once in 3–7 d	110	48⋅2	
Drank *tella* both during last pregnancy and lactation	158	69⋅3	
Main reason for mother's *tella* intake			
Taken as a food	66	29⋅0	
Fills our blood	93	40⋅8	
Helps for breast milk production	62	27⋅2	
To clean the uterus after delivery	3	1⋅3	
To quench thirst	4	1⋅7

**Table 2.** Child demographics, feeding and health characteristics in northern Ethiopia, 2019 (*n* 228)

Variable	Frequency (*n*)	Percentage (%)	Mean (sd)
Age, months			27⋅2 (5⋅2)
Sex			
Female	108	47⋅4	
Male	120	52⋅6	
Stunted	96	42⋅1	
Underweight	56	24⋅6	
Wasted	33	14⋅5	
HAZ			−1⋅87 (1⋅44)
WAZ			−1⋅29 (1⋅23)
WHZ			−0⋅41 (1⋅42)
Complementary feeding began			
At 6 months	101	44⋅3	
Before or after 6 months	127	55⋅7	
Child's usual consumption of ASF^a^			
At least once in a week	69	30⋅3	
At least once in 2–4 weeks	64	28⋅0	
For holidays only	95	41⋅7	
Child morbidity			
Child had diarrhoea at least once during last 3 months	114	50⋅0	
Child was ill at least once during the last 1 month	207	91⋅0	
Child has ever drunk *tella*	165	72⋅4	
Age child started to drink *tella*			
12 months and above	139	60⋅9	
6–12 months	22	9⋅7	
Before 6 months	4	1⋅8	
Child had *tella* while mother was drinking			
Always	104	45⋅6	
Sometimes	82	36	
Not at all	42	18⋅4	
Stunted children among mothers who consume *tella* during pregnancy	87	48⋅6	
Stunted children among mothers who did not consume *tella* during pregnancy	9	18⋅4	
Stunted children among mothers who consume *tella* during lactation	76	41⋅3	
Stunted children among mothers who did not consume *tella* during lactation	20	45⋅5	
Stunted children among mothers who consume *tella* both during pregnancy and lactation	76	48⋅1	

a ASF = meat, milk, eggs and fish.

### *Tella* intake during pregnancy and lactation

A large proportion (66⋅7 %) of mothers reported *tella* intake during the 24 h before the survey (Table 1). Among all mothers in the study, 25⋅4 % consumed two or more glasses of *tella* (total ≥500 ml) the day before the survey. During their most recent pregnancy, 78⋅5 % of mothers reported drinking some amount of *tella*; 41⋅2 % of all mothers had consumed *tella* at least once in 2 d and 37⋅3 % at least once in 3–7 d. Only 21⋅5 % of mothers reported no *tella* consumption during their last pregnancy.

Similarly, 81 % of all mothers had consumed *tella* during lactation; nearly 33 % of all mothers reported consuming at least once in 2 d while an additional 48 % consumed *tella* at least once in 3–7 d (Table 1). About 29 % of participants reported *tella* was consumed as food and 27 % said it helped for breast milk production.

Nearly half (48⋅6 %) of children whose mothers consumed *tella* during pregnancy were stunted, while only 18⋅4 % of children whose mothers did not consume *tella* during pregnancy were stunted (Table 2). However, although 41⋅3 % of children whose mothers consumed *tella* during lactation were stunted, 45⋅5 % of children whose mothers did not consume *tella* during lactation also were stunted. Also, 48⋅1 % of children from mothers who consumed *tella* both during pregnancy and lactation were stunted. Consistent with the quantitative findings, most KIs confirmed that *tella* is a socially acceptable drink used commonly by the community members. Particularly ‘*Korefe*’, a special type of *tella*, is prepared purely from barley and is widely accepted as nutritious for mothers and children. Furthermore, in addition to the perceived nutritional benefits, KIs agreed that some of the community members drank *tella* because they expected it would promote adequate breast milk production. A majority of KIs reported that a few community members drank *tella* with a belief that it cleanses the uterus after birth and estimated that most households used up to one-fourth of their household food grains for *tella* production.

### *Tella* intake by the index child

Approximately 72 % of children had consumed some amount of *tella* at least once in their life (Table 2). During 6–12 months of age, 9⋅7 % of children started to taste *tella* and an additional 60 % started to drink it at the age of ≥1 year. Most of the KIs agreed that regardless of age most mothers put a drop of *tella* in the young infant's mouth during drinking events and offered *tella* with a cup at the age of ≥1 year.

### Child feeding and morbidity characteristics

Only 44⋅3 % of mothers began complementary food for their child at the age of 6 months; others began either before or after that age (Table 2). More than 40 % of children were fed ASF only for holidays (six to seven times per year). Regarding the morbidity status of children, 91 % had a history of illness at least once in the month before the survey, and 50 % of them had at least one episode of diarrhoea within 3 months before the survey.

### Factors associated with stunting

In the adjusted logistic regression model (Table 3), maternal *tella* consumption every day or two during pregnancy, the number of children under-5 years in the household, sex and the family size were significantly associated with stunting. The odds of being stunted were increased by nearly five times (AOR 4⋅97, 95 % CI 2⋅20, 11⋅25) among children born of mothers who reported consuming *tella* at least once in 2 d during their recent pregnancy. Being male increased the likelihood of stunting by more than two times (AOR 2⋅31, 95 % CI 1⋅27, 4⋅19) compared to females. The odds of being stunted increased by more than threefold if the household had >2 under-5-year-old children (AOR 3⋅52, 95 % CI 1⋅49, 8⋅33). Furthermore, the probability of being stunted approached two times higher among children living in a family of five or more (AOR 1⋅84, 95 % CI 1⋅01, 3⋅36).

**Table 3.** Results of bivariate and multivariable logistic regression models showing the risk factors of child stunting, northern Ethiopia, 2019 (*n* 228)

Variables	COR 95 % CI	*P*-value	AOR 95 % CI	*P*-value
Age of child				
24–36 months	2⋅82 (1⋅38, 5⋅77)	0⋅004**	1⋅95 (0⋅89, 4⋅26)	
12–23 months^a^	1		1	
Sex				
Male	2⋅51 (1⋅45, 4⋅32)	0⋅001**	2⋅31 (1⋅27, 4⋅19)	0⋅006**
Female^a^	1		1	
Family size				
≥5	2⋅02 (1⋅17, 3⋅48)	0⋅011*	1⋅84 (1⋅01, 3⋅36)	0⋅045*
<5^a^	1		1	
Number of children under 5 years of age				
>2	3⋅46 (1⋅59, 7⋅52)	0⋅002**	3⋅52 (1⋅49, 8⋅33)	0⋅004**
≤2^a^	1		1	
Age complementary feeding began				
Before or after 6 months	2⋅19 (1⋅27, 3⋅77)	0⋅005**	1⋅27 (0⋅67, 2⋅40)	
At 6 months	1		1	
Child's usual consumption of ASF^b^				
Holidays only	2⋅37 (1⋅24, 4⋅53)	0⋅009**	1⋅94 (0⋅93, 4⋅04)	
Once every 2–4 weeks	1⋅28 (0⋅62, 2⋅62)	0⋅497	0⋅79 (0⋅35, 1⋅81)	
Once a week^a^	1		1	
Frequency of *tella* intake during pregnancy				
At least once in 2 d	4⋅74 (2⋅19, 10⋅2)	<0⋅001**	4⋅97 (2⋅20, 11⋅25)	<0⋅001**
At least once in 3–7 d	1⋅43 (0⋅64, 6⋅17)	0⋅373	1⋅20 (0⋅51, 2⋅81)	
Never^a^	1		1	

AOR, adjusted odds ratio; COR, crude odds ratio.

a Reference categories.

b ASF = meat, milk, eggs and fish.

* Statistically significant *P* < 0⋅05.

** Statistically significant *P* < 0⋅001

### Factors associated with underweight

The child having *tella* during each maternal drinking event, male sex and ≥3 diarrhoeal episodes within the 3 months before the survey (all *P* < 0⋅001), as well as being in the age group of 24–36 months (*P* < 0⋅05) were significantly associated with underweight (Table 4). The odds of being underweight were nearly threefold higher among children in the age groups of 24–36 months (AOR 2⋅98, 95 % CI 1⋅09, 8⋅13). The likelihood of being underweight was more than three times higher among male children (AOR 3⋅73, 95 % CI 1⋅73, 7⋅94). The odds of being underweight were more than eleven times higher among children who had three or more episodes of diarrhoea during 3 months before the survey (AOR 11⋅83, 95 % CI 4⋅22, 33⋅14). Furthermore, the likelihood of being underweight was more than fourfold higher among children who had *tella* during each maternal drinking event (AOR 4⋅23, 95 % CI 1⋅99, 8⋅97). Because the prevalence of wasting was 14⋅5 %, the sample size was too small to allow further analyses of factors associated with wasting.

**Table 4.** Results of bivariate and multivariable logistic regression models showing the risk factors of child underweight, northern Ethiopia, 2019 (*n* 228)

Variables	COR 95 % CI	*P*-value	AOR 95 % CI	*P*-value
Age of child				
24–36 months	2⋅33 (0⋅88, 9⋅53)	0⋅055	2⋅98 (1⋅09, 8⋅13)	0⋅033*
12–23 months^a^	1		1	
Sex				
Male	2⋅51(1⋅45, 4⋅32) ** (2⋅04, 8⋅13)	<0⋅001**	3⋅73 (1⋅73, 7⋅94)	<0⋅001**
Female^a^	1		1	
Family size				
≥5	1⋅96 (1⋅04, 3⋅71)	0⋅037*	1⋅77 (0⋅84, 3⋅74)	
<5^a^	1		1	
Child's usual consumption of ASF^b^				
Holidays only	2⋅34 (1⋅04, 5⋅23)	0⋅038*	2⋅01 (0⋅80, 5⋅04)	
Once every 2–4 week	2⋅49 (1⋅05, 5⋅87)	0⋅037*	1⋅73 (0⋅64, 4⋅63)	
Once a week^a^	1		1	
Age complementary feeding began				
Before or after 6 months	2⋅44 (1⋅27, 4⋅68)	0⋅007**	1⋅51 (0⋅69, 3⋅28)	
At 6 months^a^	1		1	
Frequency of diarrhoea in last 3 months				
Three or more episodes	9⋅42 (3⋅92, 22⋅6)	<0⋅001**	11⋅83 (4⋅22, 33⋅14)	<0⋅001***
One or two episodes	2⋅00 (0⋅96, 4⋅16)	0⋅062	1⋅82 (0⋅83, 3⋅98)	
No diarrhoeal episode^a^	1		1	
Child had *tella* while mother was drinking				
Always	3⋅39 (1⋅78, 6⋅42)*	<0⋅001**	4⋅23 (1⋅99, 8⋅97)	<0⋅001**
Sometimes or not at all^a^	1		1	

AOR, adjusted odds ratio; COR, crude odds ratio.

a Reference categories.

b ASF = meat, milk, eggs and fish.

* Statistically significant *P* < 0⋅05.

** Statistically significant *P* < 0⋅01.

## Discussion

Our findings showed a significant association of child undernutrition among mothers with frequent consumption of *tella* during pregnancy, and similar findings was reported with previous studies^(25,26)^. The high prevalence of stunting and underweight in the present study was also consistent with previous studies from Ethiopia^(27,28)^. Our data demonstrated that the main factors predicting child undernutrition were sex, age of the child, number of under-5-year-old children in the family, family size, maternal *tella* intake during pregnancy, frequency of diarrhoeal episodes and frequently offering the child *tella* during each drinking event. Some of these risk factors are consistent with previously identified factors such as being male and being more than 12 months old^(29)^, larger family size^(30)^, frequent diarrhoeal episodes^(31)^ and frequent intake of alcohol during pregnancy^(32,33)^. As noted by a prior study, prenatal alcohol exposure was associated with growth restrictions in weight and height, which is largely determined by birth. These growth restrictions are a particular problem among children born to women with alcohol abuse and/or dependence^(34,35)^. This may be because alcohol passes the placental barrier and allows alcohol to reach the fetus. This causes vascular constriction which compromises placental blood flow and contributes to intrauterine growth retardation and childhood stunting. Although we have not found published data in Ethiopia or elsewhere to compare our findings regarding the effect of alcohol intake by infants and young children on growth, the present study found that a majority of children start to taste *tella* at an early age. We suggest that this may exacerbate child growth restriction in the study setting.

The prevalence of alcohol (*tella*) intake by mothers during pregnancy was high (78⋅5 %) compared with previous reports from southern (8⋅1 %)^(17)^ and northern (16⋅1 %) Ethiopia^(16)^.

The significant association of frequent *tella* drinking events during pregnancy with stunting in the present study was consistent with a study in New Zealand, which assessed maternal alcohol intakes before and during pregnancy and reported their association with child growth restriction among children^(4)^. Another study also demonstrated the association of prenatal alcohol exposure with intrauterine growth restriction and suggested that this effect might persist well into early adulthood^(33)^.

In the present study, more than 80 % of mothers drank *tella* during lactation for a variety of reasons including optimising breast milk production. This is consistent with a finding reported by a recent review article, Popova and colleagues^(35)^. However, a systematic review has indicated that alcohol consumption resulted in a decrease in total breast milk yield^(36)^. Furthermore, studies demonstrate that maternal alcohol consumption may slightly reduce milk. Because some amount of alcohol consumed by a lactating woman is transferred to her milk the infant is thus exposed to alcohol. This exposure may play a negative role in infant development during this critical period^(37)^. In line with this report, the present study identified that children who had *tella* during each maternal drinking event were more than fourfold more likely to be underweight than those who never had *tella*. This may be due to the effects of alcohol on decreased appetite, digestion and nutrient uptake. However, we are not aware of studies in Ethiopia or elsewhere regarding the effect of drinking *tella* or other alcoholic beverages by the child at an early age.

The qualitative findings from the present study showed that despite some disparity among the KIs on the frequency of *tella* production at the household level and the number of food crops used by the household, the majority of KIs agreed that most households used up to one-fourth of household food grains for *tella* production and most households produced *tella* at least once in a month. The number of household food grains used for *tella* production may exacerbate the existing food insecurity in the study area. Utilisation is one dimension of food security^(38)^; hence, a strategy to advance food and nutritional security must consider utilisation rather than just producing more food^(39)^. In this regard, utilising a portion of household food crops for *tella* production may be a neglected factor for food shortage in a family, particularly in food-insecure areas. Adequate consideration by all nutrition actors and further study is required to quantify its effect.

Even though *tella* consumption is accepted by the community, the amount and frequency of drinking may be under-reported by the study participants because of the possible social desirability and recall bias during the interview. Similarly, retrospective estimation of the quantity and frequency of *tella* consumed is difficult due to the lack of common glass sizes and differences in the frequency of drinking events. Additionally, a causal relationship between the mentioned factors and the nutritional status of children cannot be inferred because of the cross-sectional design of the study which remains a limitation of the study.

## Conclusion

In conclusion, the present study confirmed previous findings on associations among male sex, family size, frequent diarrhoea and alcohol intake during pregnancy with child undernutrition. The prevalence of alcohol intake during pregnancy and lactation was higher compared with prior studies in Ethiopia. Even though the relationship may not be direct, the present study highlighted the negative nutritional implications of maternal *tella* intake on the growth of children. This implies the need for programmes to address the effects of alcohol among pregnant and lactating women. Furthermore, the possible effects of household *tella* production on food insecurity should also be emphasised.

The present study confirmed that *tella* is a socially acceptable alcoholic drink in the study area, suggesting that a strategy to reduce the effects of *tella* in the area will require thoughtful work considering the beliefs and culture of the community. Mass media campaigns and appropriate behavioural change communication models are recommended as strategies for intervention in alcohol consumption during pregnancy and lactation in the study area and similar settings.

More research on the effect of *tella* intake on fetal growth and neurocognitive development, as well as quantifying the effect of *tella* production on food insecurity is recommended. This work addresses issues of maternal alcohol intake which were inadequately considered in the past and provides baseline data for further investigation of this traditional fermented alcoholic product.
